# Impact of Moderate Heat, Carvacrol, and Thymol Treatments on the Viability, Injury, and Stress Response of *Listeria monocytogenes*

**DOI:** 10.1155/2015/548930

**Published:** 2015-10-11

**Authors:** L. Guevara, V. Antolinos, A. Palop, P. M. Periago

**Affiliations:** ^1^Departamento de Ingeniería de Alimentos y del Equipamiento Agrícola, Campus de Excelencia Internacional Regional “Campus Mare Nostrum”, Escuela Técnica Superior de Ingeniería Agronómica, Universidad Politécnica de Cartagena, Paseo Alfonso XIII 48, Cartagena, 30203 Murcia, Spain; ^2^Instituto de Biotecnología Vegetal, Campus de Excelencia Internacional Regional “Campus Mare Nostrum”, Universidad Politécnica de Cartagena, Edificio I+D+I, Muralla del Mar, Cartagena, 30202 Murcia, Spain

## Abstract

The microbial safety and stability of minimally processed foods are based on the application of combined preservative factors. Since microorganisms are able to develop adaptive networks to survive under conditions of stress, food safety may be affected, and therefore understanding of stress adaptive mechanisms plays a key role in designing safe food processing conditions. In the present study, the viability and the sublethal injury of *Listeria monocytogenes* exposed to moderate heat (55°C) and/or essential oil compounds (carvacrol and thymol, 0.3 mM) treatments were studied. Synergistic effects were obtained when combining mild heat (55°C) with one or both essential oil compounds, leading to inactivation kinetics values three to four times lower than when using heat alone. All the treatments applied caused some injury in the population. The injury levels ranged from around 20% of the surviving population under the mildest conditions to more than 99.99% under the most stringent conditions. Protein extracts of cells exposed to these treatments were analysed by two-dimensional gel electrophoresis. The results obtained revealed that stressed cells exhibited differential protein expression to control cells. The proteins upregulated under these stressing conditions were implicated, among other functions, in stress response, metabolism, and protein refolding.

## 1. Introduction

*Listeria monocytogenes* is a Gram-positive foodborne pathogen microorganism. This microorganism causes a disease called listeriosis, which has been associated with outbreaks by ingestion of milk, cheese, vegetables, salads, and meat [1, 2]. The pervasiveness of this microorganism is due, in part, to its ability to tolerate extreme environment conditions (high salt concentration, wide range of pH and temperature, and low water availability) [3].

Food manufactures and consumers demand additive-free, fresher, and full tasting food products while maintaining high standards of microbiological safety. The use of natural antimicrobial systems for preservation of foods could accomplish this demand. Although essential oil components are used as flavourings in the food industry, nowadays they represent a highly interesting source of natural antimicrobials for food preservation due to their antimicrobial and antioxidative activity [4]. Carvacrol and thymol are natural phenolic compounds present in the essential oil fraction of* Origanum* and* Thymus* plants [5, 6] and have long been used in foods as flavour enhancers. Both compounds of essential oils (carvacrol and thymol) have been shown to exhibit antibacterial and antifungal activity including food pathogens [5–10]. A synergistic effect of nisin, carvacrol, and thymol [9, 11] or thymol and cymene [7, 12] against vegetative cells of* B. cereus* has been observed. When a mild thermal treatment was applied prior to the growth in presence of antimicrobials, the sensitivity to antimicrobials was increased [8, 11]. Likewise, a synergistic effect of carvacrol and cymene [13] or nisin and carvacrol [14] to control growth and viability of* Listeria monocytogenes* has also been shown. Again, heat was able to increase* L. monocytogenes* sensitivity to antimicrobials [14]. The antimicrobial property of carvacrol and thymol has been attributed to the considerable effects on the structural and functional properties of cytoplasmatic membrane [15]. Cell membrane alterations caused by these compounds are able to induce sublethal injury.

As sublethal injury is supposed to be related to the higher sensitivity of survivors to stress conditions after treatment, the success of a combined treatment should be correlated with the degree of sublethal injury caused by the hurdles in the bacterial population [16]. Moreover, under suitable conditions, sublethal injured cells might be repaired, which is a very important aspect to be taken into account regarding food safety. A few cells being capable of repairing damage after moderate heat and essential oil compounds treatment could result in infective concentrations.

The microbial safety and stability of most minimally processed foods are based on application of combined preservative factors of which (mild) heating is the most common preservation technique in use these days. Bacteria have evolved adaptive networks to face the challenges of changing environments and to survive under conditions of stress [17]. When bacterial cells, grown at an optimal temperature, are shifted to a higher temperature a heat-shock response develops and heat resistance and the synthesis of a set of heat-shock proteins (HSPs) are induced. Bacterial heat resistance is affected by a wide variety of genetic, physiological, and environmental factors. When microorganisms develop resistance to commonly used preservation methods, food quality and safety may be affected, and therefore understanding of stress adaptive mechanisms plays a key role in designing safe food processing conditions [18].

So far, the combined effect of carvacrol, thymol, and mild heat treatments on the viability, sublethal injury, and the protein expression profile of* L. monocytogenes* cells is not well known. Therefore, in the present study, the viability and the sublethal injury of cells of* L. monocytogenes* CECT 4031 exposed to moderate heat and/or essential oils compounds (carvacrol and thymol) treatments were studied. Then, protein extracts of cells exposed to these treatments were analysed by two-dimensional gel electrophoresis (2D-electrophoresis) and 20 proteins were identified and their roles were discussed.

## 2. Materials and Methods

### 2.1. Bacterial Strains and Preparation of Inoculum

*L. monocytogenes* CECT 4031, provided by the Spanish Type Culture Collection (CECT, Valencia, Spain), was used in the present study. Cells were grown overnight at 37°C in Tryptic Soy Broth (TSB; Scharlau, Barcelona, Spain) containing 0.6 g/100 g yeast extract (TSBYE; Scharlau). Then,* L. monocytogenes* was inoculated into fresh medium (TSBYE) and incubated at 37°C with shaking at 140 rpm until the stationary phase was reached.

### 2.2. Chemicals

Carvacrol (Fluka Chemie AG, Buchs, Switzerland) and thymol (Sigma Aldrich Chemie, Steinheim, Germany) stock solutions were held in 95% ethanol at 4°C.

### 2.3. Effect of Mild Heat Treatment and/or Essential Oils Compounds (Carvacrol and Thymol) on* L. monocytogenes* Cells Viability

Cultures in stationary phase, grown at 37°C, were harvested and concentrated by centrifugation (3500 ×g, 10 min, at 4°C) and resuspended in TSBYE. Treatments with only essential oil compounds were carried out through exposures of* L. monocytogenes* to carvacrol alone (0.3 mM), thymol alone (0.3 mM), or both compounds together (0.3 mM of each one) in 5 mL TSBYE medium (initial concentration of* L. monocytogenes* 10^10^ CFU/mL) at 25°C during 30 min in a water bath. These concentrations of antimicrobials did not modify the sensorial properties and only when combined led to slight changes in odour. Control experiments with the same concentration of ethanol (without essential oils) showed a stable viable count over 30 min of incubation in all cases (data not shown). For mild heat treatments, preheated 5 mL TSBYE medium (initial concentration of* L. monocytogenes* 10^10^ CFU/mL) kept at 55°C in a water bath was used. Mild heat treatments alone (55°C) and combined with essential oil compounds (55°C and carvacrol 0.3 mM; 55°C and thymol 0.3 mM; 55°C and carvacrol 0.3 mM and thymol 0.3 mM) during 30 min were carried out. Viable counts were analysed after 0, 5, 10, 15, 20, and 30 min of exposure. For all treatments three independent experiments were performed and samples (whose tenfold serial dilutions were prepared in buffered peptone water (Scharlau)) were plated, in duplicate, by the pour plate method on Tryptic Soy Agar (TSA, Scharlau) containing 0.6 g/100 g yeast extract (TSAYE). The plates were incubated for 24–48 h at 37°C. Survival curves were plotted as the logarithm of CFU/mL versus exposure time.

### 2.4. Determination of Degree Injury: Enumeration of Viable and Injured Cells

To determine the loss of viability caused by a treatment, untreated and treated cell suspensions were serially diluted and plated on the surface of an appropriate count medium. TSAYE was used as nonselective agar medium in the enumeration of viable* L. monocytogenes*. Sublethal injury of* L. monocytogenes* exposed to mild heat and/or essential oils compounds treatments was assessed by the difference between the counts on the nonselective agar medium (TSAYE) and the selective agar medium, TSAYE, supplemented with NaCl 5% (wt/vol) (Panreac, Spain) (TSAYE-SC). 5% was the maximum noninhibitory sodium chloride concentration for native cells previously determined for* L. monocytogenes* [19]. The plates were incubated for 24–48 h at 37°C. The data were presented as means of at least three independent experiments. Percent of injury was calculated for each tripled sample using the following equation [20]:

(1) $$\begin{array}{c} \%\;\;\text{injury}=100\times \frac{{cfu}_{\text{TSAYE}}-{cfu}_{\text{TSAYE-}SC}}{{cfu}_{\text{TSAYE}}}. \end{array}$$

### 2.5. Modelling of Survival Curves

Survival curves of* L. monocytogenes* after exposure to treatments (mild heat and/or essential oils compounds) were drawn by plotting the log of microorganisms against exposure time. These curves were fitted using the cumulative function of the Weibull distribution as proposed by Mafart et al. [21] to describe the inactivation of microorganisms in terms of decimal logarithms:

(2) $$\begin{array}{c} \log_{10}N_{t}=\log_{10}N_{0}-{\left(\;\frac{t}{\delta }\;\right)}^{p}, \end{array}$$

where *t* is the treatment time, *N*_*t*_ and *N*_0_ are the population densities at time *t* and time zero, respectively, *δ* is the scale parameter (time to inactivate the first logarithmic cycle of microbial population), and *p* is the shape parameter, which describes the behavior of the population: if *p* < 1 an upward concavity is happening, if *p* > 1 a downward concavity will be observed, and if *p* = 1 the survival curve is linear.

Since all survival curves had the same tail-shape, a single *p* value for all of them was used as proposed by Couvert et al. [22].

Data were analyzed using Statgraphics Plus 5.1 software (Statistical Graphics Corp., Rockville, MD). One-way analysis of variance (ANOVA) for the parameters derived from the survival experiments was used to establish significant differences between the different treatments.

### 2.6. Effect of Mild Heat Treatment and/or Essential Oils Compounds (Carvacrol and Thymol) on* L. monocytogenes* Protein Expression

#### 2.6.1. Differential Proteome Analysis of* L. monocytogenes* Cells

Total cellular protein extractions were performed as described by Wouters et al. [23]. For each sample, 10 mL of stationary phase cultures of* L. monocytogenes *STCC4031 (control), exposed to mild heat alone (55°C) and combined with essential oil compounds (55°C and carvacrol 0.3 mM and thymol 0.3 mM) during 30 min, was centrifuged at 4,000 rpm during 10 minutes. The pellets were resuspended in 1 mL of lysis buffer (urea 8 M, CHAPS 2% (w/v), protease inhibitor (Roche, Germany), and DTT 5 mM). Consecutively, cells were disrupted by bead beating with a MiniBead Beater cell homogenizer (Biospec Products, Bartlesville, OK) and zirconium beads (0.1 mm diameter; Biospec Products) six times for 1 min (with cooling on ice between treatments). The protein concentration in cell-free extracts was determined using the kit RC-DC protein assay (Bio-Rad). Samples of homogenate were stored at −80°C.

#### 2.6.2. Two-Dimensional Gel Electrophoresis

2D-electrophoresis was performed, as described [24, 25], using gradient Immobiline Dry Strips pH 4–7 (7 cm; Amersham Biosciences) for the first dimension (IEF). Proteins samples (20 *μ*g) were carried to a 125 *μ*L dehydration buffer (7 M urea, 2 M thiourea, 4% CHAPS, 0.5% IPG buffer (pH 4), 50 mM DTT, and bromophenol blue traces) and loaded onto the IPG strip. Isoelectric focusing was performed in IPGPhor-I (Amersham Biosciences) to reach 6500 Vh in total. After the separation, the first dimension strips were equilibrated twice with equilibration buffer (6 M urea, 50 mM Tris–HCl pH 8.8, 20% glycerol, and 2% SDS) in the presence of 1% DTT in the first equilibration and 4% iodoacetamide in the second one. SDS-PAGE in the second dimension was performed in 12% polyacrylamide home-casted gels. Gels were run in a Mini-Protean System (Bio-Rad) until the tracking dye reached the bottom of the gel.

#### 2.6.3. Gel Staining and Image Analysis

After electrophoresis, the gels were stained with MALDI TOF compatible silver nitrate using a Plus One Silver Staining Kit (Amersham Biosciences), as described by Shevchenko et al. [26]. Gels were scanned with an ImageScanner II Desktop (Amersham Biosciences) and the image analysis of the gels was performed using PD Quest 8.0.1 software (Bio-Rad). Three gels were produced from independent cultures of each condition and representative image gels are shown. Induction factors for each induced protein were calculated as the ratio between the normalized value in “treated” gel and the normalized value in “control” gel. Protein spots displaying ≥2-fold change in abundance were chosen for analysis by mass spectrometry.

#### 2.6.4. Protein Identification

Spots were manually excised from stained gels and sent for the digestion, the analysis by MS/MS, and the database searching to the Proteomics Lab of the Centro Nacional de Biotecnología (CNB-CSIC, Madrid, Spain). The digestion protocol used was based on Shevchenko et al. [26]. The MS/MS analysis was carried out in a MALDI-TOF/TOF (4800 Plus MALDI-TOF/TOF Analyzer, Applied Biosystems). Measured tryptic peptide mass values were transferred using the MS Bio Tools (Bruker) for searching in the National Center for Biotechnology Information (NCBI) nonredundant database using Mascot software (http://www.matrixscience.com/; Matrix Science, London, UK) as search engine. The confidence interval for protein identification was set to ≥95% (*p* < 0.05) and only peptides with a minimum ion score of 79 were considered correctly identified.

## 3. Results and Discussion

### 3.1. Effect of Mild Heat Treatment and/or Essential Oil Compounds (Carvacrol and Thymol) on* L. monocytogenes* Cells Viability

*L. monocytogenes *cells were sensitive to all treatments applied, either heat or antimicrobials, individually or combined (Table 1). All survival curves showed a tail-shape, so the use of classical lineal models, such as the Bigelow model, was not recommended, and the nonlinear Weibull model was used instead in order to fit the survival data. Since all the survival curves showed approximately the same shape, a single *p* value (*p* = 0.41) was used to fit all the survival curves, as proposed by Couvert et al. [22].

**Figure 1:**
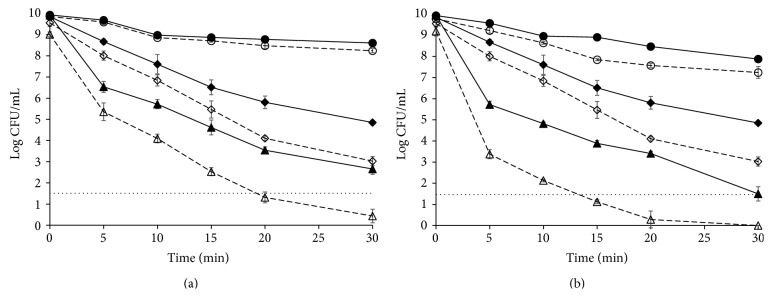
Survival curves of* Listeria monocytogenes* CECT 4031 obtained in TSBYE and recovered in TSAYE (continuous lines) or TSAYE-SC (dashed lines). (a) In presence of 0.3 mM thymol (●) and at 55°C (◆) and at 55°C and in presence of 0.3 mM thymol (▲). (b) In presence of 0.3 mM thymol + 0.3 mM carvacrol (●) and at 55°C (◆) and at 55°C and in presence of 0.3 mM thymol + 0.3 mM carvacrol (▲). Horizontal dotted lines show the counting technique detection limit.

Both thymol and carvacrol inactivated about one log cycle of the initial population of* L. monocytogenes *cells within the first 20 min of exposition, showing *δ* values of 15.58 and 12.79 min, respectively, when these antimicrobials were added to the treatment medium (Table 1).  Figure 1(a) shows, as an example, the survival curve of* L. monocytogenes *exposed to 0.3 mM thymol.

When both antimicrobials were added together, approximately additive results were reached, since one log cycle was inactivated within the first 10 min of exposition and a *δ* value of 8.74 min was obtained (Table 1 and Figure 1(b)).

Mild heat (55°C) was much more effective in inactivating* L. monocytogenes *cells, since a *δ* value of 0.80 min was obtained and 5 log cycles of inactivation were reached after 30 min of treatment (Figure 1 and Table 1).

Combinations of heat with either thymol or carvacrol led again to synergistic results with *δ* values of 0.25 min in both cases (Table 1). Also the combination of all heat and thymol and carvacrol led to synergistic results with a *δ* value of 0.18 min and more than 8 log cycles' inactivation within 30 min (Figure 1(b) and Table 1).

When antimicrobials and heat are combined, synergistic results are usually obtained [27–30] with heat resistance kinetics parameters being reduced down to three times [31–33]. In our case, *δ* values were reduced from 0.80 min when a thermal treatment at 55°C was applied to 0.25 min when either thymol or carvacrol was added to the heating medium, which is within the average reductions. When both antimicrobials were added together to the heating medium, further reductions were obtained (0.18 min, i.e., more than four times).

### 3.2. Effect of Mild Heat Treatment and/or Essential Oils Compounds (Carvacrol and Thymol) on the Level of Injury

All the treatments applied caused some injury in the population of* L. monocytogenes* cells, as assessed by growth of the survivors in presence of NaCl (Figure 1 and Table 2). Injury levels ranged from very low when only thymol or carvacrol was applied for short exposition times (around 20% of the population was injured after 5 min exposition to thymol, Figure 1(a)) to more than 99% of the population where more stringent conditions were applied (Table 2). Heat caused more injury than antimicrobials when applied individually (Figure 1), since only 5 min at 55°C was sufficient to cause injury in more than 75% of the population (Table 2). Injured population increased as the thermal treatment proceeded. When heat and antimicrobials were combined, injured population was higher than 90% from the first moments of the treatment.

The synergistic effects of combined heat and antimicrobials have been explained in terms of inactivation of heat-injured cells when the antimicrobials are present in the heating medium [33]; that is, heat causes injuries to different cell structures but is not able to inactivate these injured cells when applied alone. However, when combined, antimicrobials would help to inactivate these injured cells. In our case, the synergistic effects of combining heat and antimicrobials could also be explained as a consequence of the inactivation of heat-injured cells by antimicrobials, since the inactivation curve of* L. monocytogenes *exposed to a mild thermal treatment and recovered in presence of NaCl is very close to that of cells exposed to a combined heat-thymol treatment and plate counts even overlap after 30 minutes of exposure (Figure 1(a)). Similar results were obtained when using carvacrol instead of thymol.

However, in our case, when heat was combined with antimicrobials additional injured population was shown, as evidenced by recovery in presence of NaCl of the survivors of this combined treatments (Table 2 and Figure 1). Hence, even when antimicrobials could help to inactivate the population injured by heat, a new injured population appeared after the combined treatment.

### 3.3. Effect of Mild Heat Treatment and/or Essential Oils Compounds (Carvacrol and Thymol) on* L. monocytogenes* Protein Expression

**Figure 2:**
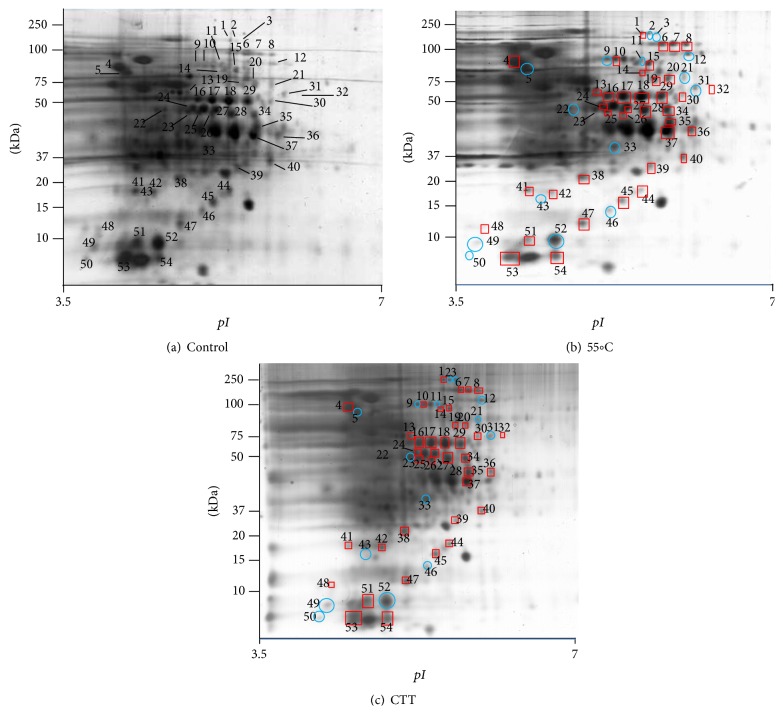
2D-electrophoresis of extracts of stationary phase* Listeria monocytogenes* CECT 4031 grown in TSAYE at 37°C: (a) resuspended in TSAYE; (b) exposed to 55°C; (c) exposed to 55°C in presence of 0.3 mM thymol + 0.3 mM carvacrol, for 30 minutes. Induced proteins (squares) and repressed proteins (circles) are numerated (see also Tables 3 and 4).

To gain an overview of the proteins induced upon mild heat treatment alone (55°C) and combined with essential oils compounds (55°C and carvacrol 0.3 mM and thymol 0.3 mM), 2D-electrophoresis was used. On gels containing cell-free extracts of control and treated/stressed* L. monocytogenes *cells, a total of approximately 200 spots could be detected (Figure 2). Interestingly, comparative proteomic analysis between treated and untreated* L. monocytogenes* cells showed that 54 spots exhibited different induction levels of twofold or more at least in one of the treatments applied (Table 3 and Figure 2). 38 proteins were upregulated and 16 downregulated twofold or more at least in one of the stresses applied (Table 3). The same 38 proteins were upregulated when any of the stress treatments were applied but showing different induction levels. The same happened with the 16 downregulated proteins. Surprisingly, although in our study the combined treatment (heat with both antimicrobial compounds) presented a higher antimicrobial effect on the viability of* L. monocytogenes* than heat alone, the combined treatment did not show, in general, a stronger protein response. Neither mild heat treatment alone nor mild heat treatment combined with essential oil compounds showed a specific trend in protein induction levels. Among the overexpressed protein spots, those upregulated more than twofold, after exposition either to 55°C or to 55°C and carvacrol 0.3 mM and thymol 0.3 mM, were excised from the gels and subjected to MALDI-TOF MS/MS analysis for the protein identification and determination of their biological functions. 20 proteins were successfully identified out of the whole set of overexpressed protein spots (Table 4). Spots 18, 20, 28, 32, 34, 35, 37, 38, 41, 44, and 45 were identified as glyceraldehyde-3-phosphate dehydrogenase (GAPDH), 3-bisphosphoglycerate-independent phosphoglycerate mutase, lactate dehydrogenase, triosephosphate isomerase, rod shape-determining protein MreB, PTS mannose transporter subunit IIAB, cysteine synthase, ATP-dependent Clp protease proteolytic subunit, transcription elongation factor GreA, hypothetical protein lmo2511, and 50 ribosomal protein L10, respectively. Spot 24 yielded two different proteins that were identified as translation elongation factor Ts and molecular chaperone DnaK and spot 47 yielded another two proteins identified as hypothetical protein lmo1580 and universal stress protein. Likewise, spot 51 showed that it contains two different proteins: regulatory protein SpoVG and 50S ribosomal protein L7/L12. Finally, spot 53 contained three different proteins: cochaperonin GroES, major cold-shock protein homolog CspB, and 50S ribosomal protein L12. According to biological functions, the 20 identified proteins belong to different categories including mainly stress response (44, 47a, 47b, and 53b), metabolic processes and their regulation (18, 20, 28, 32, 35, and 37), protein synthesis (24a, 41, 45, 51c, and 53c), protein folding (24b, 53a), and protease activity (38) (Table 4).

The results obtained in the current study revealed that* L. monocytogenes* CECT 4031 control cells exhibited differential protein expression to stressed cells. The proteins upregulated under these stressing conditions are implicated, among other functions, in stress response, metabolism, and protein refolding.

In bacterial cells heat causes damage to macromolecular cell components such as proteins and DNA. Carvacrol and thymol show the same mechanism of action due to their similar chemical structure. Both EOs seem to increase cytoplasmic membrane permeability [15] causing the destruction of the lipid bilayer in Gram-negative bacteria. As a consequence, there is an efflux of ions and ATP causing proton motive force dissipation and eventually cell death. To cope with these adverse conditions, response to heat stress involves protein chaperones, which assist in folding and assembly of heat damaged proteins [34] and proteases.

In our study, the treatment applied induced the overexpression in* L. monocytogenes* of the chaperone DnaK (spot 24b, Table 4), the cochaperonin GroES (spot 53a, Table 4), and ATP-dependent Clp protease subunit (spot 38, Table 4). Chaperone DnaK was also overexpressed when* L. monocytogenes *was exposed to other antimicrobial substances, such as nisin [35] or enterocin AS-48 [36]. These chaperones have been reported to be required in* L. monocytogenes* for tolerance to environmental stresses [37] and in the autolytic proteome [38]. Others studies also showed that some chaperones and proteases are heat-shock proteins (HSPs) expressed in response of* Bacillus cereus* and* Bacillus weihenstephanensis* to even moderated temperatures or other cellular stresses [39, 40].

Cell metabolism is essential for energy generation, DNA replication, and cell division. Overexpression of proteins involved in energy metabolism could be an attempt to compensate for partially impaired energy generation caused by stressing treatments interacting with the bacterial cytoplasmic membrane. In fact, our results showed that stress treatments induced the expression in* L. monocytogenes* of five proteins involved in metabolic processes, between two- and fourfold. Other authors found that proteins like glyceraldehyde-3-phosphate dehydrogenase (GAPDH) and cysteine synthase were induced in the autolytic proteome of* L. monocytogenes* [38] and exposure to nisin [35].

In our study, an overproduction of proteins of stress response, like the major cold-shock-protein CspB, was observed when treatments were applied. The same protein was reported to have been induced in* B. cereus* when exposed to stresses as heat and ethanol [40]. Interestingly, the MreB rod shape-determining protein and the regulatory protein SpoVG were also induced in our study and likewise were found in* B. cereus* exposed to moderate heat or ethanol [40] and in* L. monocytogenes* isolated from smoked mussels [41].

The phenotypic and genotypic robustness of* L. monocytogenes* is of particular concern to the food industry as it has a variety of encoded proteins implicated in survival mechanisms to withstand environmental stressors such as heat, cold, salt, acidic pH, or antimicrobials [42, 43]. In addition, some of these stress responses can result in enhanced microbial survival, enhanced thermotolerance, and even cross protection against multiple stresses [39, 40, 44].

## 4. Conclusions

Our results show that a combined treatment of moderate heat with antimicrobials (carvacrol and/or thymol) brings synergistic effects. Both heat and antimicrobials cause some degree of injury in the cells surviving moderate individual or combined treatments. Stress response in these injured cells involves overexpression of proteins implicated, among other functions, in stress response, metabolism, and protein refolding. This stress response could result in enhanced survival, compromising the safety of foods preserved by combined processes.

## Figures and Tables

**Table 1:** *δ* values (min) of *Listeria monocytogenes* CECT 4031 (*p* = 0.41) obtained in TSBYE at 55°C and/or in presence of 0.3 mM thymol and/or 0.3 mM carvacrol and recovered in TSAYE or TSAYE-SC.

	Recovery in TSAYE			Recovery in TSAYE-SC		
	*δ* value (min)	RMSE	*r* ^2^	*δ* value (min)	RMSE	*r* ^2^
Thymol	15.58	0.226	0.893	11.02	0.259	0.901
Carvacrol	12.79	0.249	0.898	11.07	0.252	0.904
Thymol + carvacrol	8.74	0.334	0.878	3.76	0.393	0.907
55°C	0.80	0.655	0.926	0.43	0.944	0.911
55°C + thymol	0.25	0.276	0.993	0.16	0.447	0.988
55°C + carvacrol	0.25	0.319	0.990	0.14	0.419	0.989
55°C + thymol + carvacrol	0.18	0.332	0.992	0.10	0.813	0.966

**Table 2:** Percentage of injury in *Listeria monocytogenes* CECT 4031 cells after different exposure times to mild heat (55°C) applied alone or in presence of 0.3 mM thymol and/or 0.3 mM carvacrol.

Time (min)	55°C	55°C + thymol	55°C + carvacrol	55°C + thymol + carvacrol
5	76.27 ± 11.60^aA^	92.76 ± 3.25^aB^	98.19 ± 0.59^aC^	99.53 ± 0.14^aD^
10	79.78 ± 12.21^aA^	97.25 ± 1.58^bB^	99.16 ± 0.72^abBC^	99.79 ± 0.06^bC^
15	90.74 ± 2.43^aA^	99.12 ± 0.36^bB^	99.44 ± 0.48^bBC^	99.82 ± 0.05^bC^
20	97.33 ± 2.58^bA^	99.24 ± 0.56^bA^	99.75 ± 0.25^bA^	99.89 ± 0.12^bcA^
30	98.39 ± 0.69^bA^	99.04 ± 1.02^bAB^	99.87 ± 0.20^bB^	≥99.99^cB^

^a–c^The same lowercase letters indicate that there are no significant differences in columns.

^A–D^The same capital letters indicate that there are no significant differences in rows.

**Table 3:** Induction factor^a^ of proteins of *Listeria monocytogenes* CECT 4031 exposed to mild heat treatment alone (55°C) and combined with essential oils compounds (55°C and carvacrol 0.3 mM and thymol 0.3 mM) for 30 minutes.

Spot number	55°C	55°C + EOs
1	2.0	2.1
2	−4.3	−14.1
3	−4.9	−4.8
4	3.2	2.4
5	−2.1	−3.9
6	3.1	2.2
7	2.7	2.9
8	2.5	2.8
9	−1.7	−2.5
10	2.1	1.6
11	−1.5	−2.1
12	−1.8	−2.0
13	8.4	6.4
14	2.0	3.3
15	1.4	2.0
16	18.5	12.7
17	11.2	9.2
18	4.5	4.3
19	2.0	1.9
20	3.5	2.1
21	−14.7	−8.8
22	−3.3	0.0
23	0.0	−12.3
24	4.2	3.9
25	2.0	2.2
26	5.1	2.0
27	2.0	2.1
28	2.0	2.2
29	6.0	5.0
30	9.1	5.3
31	−1.6	−2.7
32	2.2	2.4
33	−7.7	−3.4
34	8.5	5.0
35	9.1	5.3
36	2.1	1.6
37	2.7	1.7
38	7.8	10.8
39	2.6	2.0
40	2.0	2.6
41	1.2	2.1
42	3.3	7.6
43	−2.0	−2.2
44	2.3	2.0
45	2.0	3.3
46	−2.0	−2.0
47	18.5	12.7
48	10.2	11.2
49	−9.6	−1.6
50	−2.5	−1.6
51	2.3	2.5
52	−13.5	−7.6
53	2.0	3.1
54	2.5	2.0

^a^Normalized value in treated gel/normalized value in control gel.

**Table 4:** Identification of proteins upregulated in *Listeria monocytogenes* CECT 4031 exposed to mild heat treatment alone (55°C) and combined with essential oils compounds (55°C and carvacrol 0.3 mM and thymol 0.3 mM) for 30 minutes.

Spot number	Protein identity	Accession number	Mascot score	Biological function
18	Glyceraldehyde-3-phosphate dehydrogenase	gi/16804497	728	Metabolic processes
20	3-Bisphosphoglycerate-independent phosphoglycerate mutase	gi/441475375	141	Metabolic processes
24a	Translation elongation factor Ts	gi/47014632	262	Protein synthesis
24b	Molecular chaperone DnaK	gi/16803513	105	Protein folding
28	Lactate dehydrogenase	gi/185497273	128	Metabolic processes
32	Triosephosphate isomerase	gi/16804495	601	Metabolic processes
34	Rod shape-determining protein MreB	gi/16803588	93	Determination of bacterial cytoskeleton
35	PTS mannose transporter subunit IIAB	gi/16802144	117	Regulation of metabolic and transcriptional processes
37	Cysteine synthase	gi/16802269	191	Metabolic processes
38	ATP-dependent Clp protease proteolytic subunit	gi/16804506	187	Protease activity
41	Transcription elongation factor GreA	gi/735685227	198	Protein synthesis
44	Hypothetical protein lmo2511	gi/16804549	345	Stress response
45	50 ribosomal protein L10	gi/685938168	105	Protein synthesis
47a	Hypothetical protein lmo1580	gi/16803620	204	Stress response
47b	Universal stress protein	gi/46907811	204	Stress response
51a	Regulatory protein SpoVG	gi/16802242	180	Cell division
51b	50S ribosomal protein L7/L12	gi/16802297	110	Protein synthesis
53a	Cochaperonin GroES	gi/16804108	116	Protein folding
53b	Major cold-shock protein homolog CspB	gi/1864167	105	Stress response
53c	50S ribosomal protein L12	gi/786164	58	Protein synthesis

## References

[1] Jacquet C., Catimel B., Brosch R. (1995). Investigations related to the epidemic strain involved in the French listeriosis outbreak in 1992. *Applied and Environmental Microbiology*.

[2] Schlech W. F., Lavigne P. M., Bortolussi R. A. (1983). Epidemic listeriosis—evidence for transmission by food. *The New England Journal of Medicine*.

[3] Gandhi M., Chikindas M. L. (2007). Listeria: a foodborne pathogen that knows how to survive. *International Journal of Food Microbiology*.

[4] Burt S. (2004). Essential oils: their antibacterial properties and potential applications in foods—a review. *International Journal of Food Microbiology*.

[5] Juven B. J., Kanner J., Schved F., Weisslowicz H. (1994). Factors that interact with the antibacterial action of thyme essential oil and its active constituents. *Journal of Applied Bacteriology*.

[6] Sivropoulou A., Papanikolaou E., Nikolaou C., Kokkini S., Lanaras T., Arsenakis M. (1996). Antimicrobial and cytotoxic activities of *Origanum* essential oils. *Journal of Agricultural and Food Chemistry*.

[7] Delgado B., Fernández P. S., Palop A., Periago P. M. (2004). Effect of thymol and cymene on *Bacillus cereus* vegetative cells evaluated through the use of frequency distributions. *Food Microbiology*.

[8] Periago P. M., Conesa R., Delgado B., Fernández P. S., Palop A. (2006). *Bacillus megaterium*spore germination and growth inhibition by a treatment combining heat with natural antimicrobials. *Food Technology and Biotechnology*.

[9] Periago P. M., Moezelaar R. (2001). Combined effect of nisin and carvacrol at different pH and temperature levels on the viability of different strains of *Bacillus cereus*. *International Journal of Food Microbiology*.

[10] Ultee A., Slump R. A., Steging G., Smid E. J. (2000). Antimicrobial activity of carvacrol toward *Bacillus cereus* on rice. *Journal of Food Protection*.

[11] Periago P. M., Palop A., Fernández P. S. (2001). Combined effect of nisin, carvacrol and thymol on the viability of *Bacillus cereus* heat-treated vegetative cells. *Food Science and Technology International*.

[12] Delgado B., Palop A., Fernández P. S., Periago P. M. (2004). Combined effect of thymol and cymene to control the growth of *Bacillus cereus* vegetative cells. *European Food Research and Technology*.

[13] Periago P. M., Delgado B., Fernández P. S., Palop A. (2004). Use of carvacrol and cymene to control growth and viability of *Listeria monocytogenes* cells and predictions of survivors using frequency distribution functions. *Journal of Food Protection*.

[14] Esteban M.-D., Palop A. (2011). Nisin, carvacrol and their combinations against the growth of heat-treated *Listeria monocytogenes* cells. *Food Technology and Biotechnology*.

[15] Lambert R. J. W., Skandamis P. N., Coote P. J., Nychas G.-J. E. (2001). A study of the minimum inhibitory concentration and mode of action of oregano essential oil, thymol and carvacrol. *Journal of Applied Microbiology*.

[16] Wuytack E. Y., Phuong L. D. T., Aertsen A. (2003). Comparison of sublethal injury induced in *Salmonella enterica* serovar *Typhimurium* by heat and by different nonthermal treatments. *Journal of Food Protection*.

[17] Abee T., Wouters J. A. (1999). Microbial stress response in minimal processing. *International Journal of Food Microbiology*.

[18] Bower C. K., Daeschel M. A. (1999). Resistance responses of microorganisms in food environments. *International Journal of Food Microbiology*.

[19] García D., Gómez N., Mañas P., Condón S., Raso J., Pagán R. (2005). Occurrence of sublethal injury after pulsed electric fields depending on the micro-organism, the treatment medium pH and the intensity of the treatment investigated. *Journal of Applied Microbiology*.

[20] Muñoz M., De Ancos B., Sánchez-Moreno C., Cano M. P. (2007). Effects of high pressure and mild heat on endogenous microflora and on the inactivation and sublethal injury of *Escherichia coli* inoculated into fruit juices and vegetable soup. *Journal of Food Protection*.

[21] Mafart P., Couvert O., Gaillard S., Leguerinel I. (2002). On calculating sterility in thermal preservation methods: application of the Weibull frequency distribution model. *International Journal of Food Microbiology*.

[22] Couvert O., Gaillard S., Savy N., Mafart P., Leguérinel I. (2005). Survival curves of heated bacterial spores: effect of environmental factors on Weibull parameters. *International Journal of Food Microbiology*.

[23] Wouters J. A., Jeynov B., Rombouts F. M., de Vos W. M., Kuipers O. P., Abee T. (1999). Analysis of the role of 7 kDa cold-shock proteins of *Lactococcus lactis* MG1363 in cryoprotection. *Microbiology*.

[24] Görg A., Obermaier C., Boguth G. (2000). The current state of two-dimensional electrophoresis with immobilized pH gradients. *Electrophoresis*.

[25] Rabilloud T. (1998). Use of thiourea to increase the solubility of membrane proteins in two-dimensional electrophoresis. *Electrophoresis*.

[26] Shevchenko A., Wilm M., Vorm O., Mann M. (1996). Mass spectrometric sequencing of proteins from silver-stained polyacrylamide gels. *Analytical Chemistry*.

[27] Cava-Roda R. M., Taboada A., Palop A., López-Gómez A., Marin-Iniesta F. (2012). Heat resistance of *Listeria monocytogenes* in semi-skim milk supplemented with vanillin. *International Journal of Food Microbiology*.

[28] Char C., Guerrero S., Alzamora S. M. (2009). Survival of *Listeria innocua* in thermally processed orange juice as affected by vanillin addition. *Food Control*.

[29] Espina L., Condón S., Pagán R., García-Gonzalo D. (2014). Synergistic effect of orange essential oil or (+)-limonene with heat treatments to inactivate *Escherichia coli* O157:H7 in orange juice at lower intensities while maintaining hedonic acceptability. *Food and Bioprocess Technology*.

[30] Juneja V. K., Altuntaş E. G., Ayhan K., Hwang C.-A., Sheen S., Friedman M. (2013). Predictive model for the reduction of heat resistance of *Listeria monocytogenes* in ground beef by the combined effect of sodium chloride and apple polyphenols. *International Journal of Food Microbiology*.

[31] Char C. D., Guerrero S. N., Alzamora S. M. (2010). Mild thermal process combined with vanillin plus citral to help shorten the inactivation time for *Listeria innocua* in orange juice. *Food and Bioprocess Technology*.

[32] Cherrat L., Espina L., Bakkali M., García-Gonzalo D., Pagán R., Laglaoui A. (2014). Chemical composition and antioxidant properties of *Laurus nobilis* L. and *Myrtus communis* L. essential oils from Morocco and evaluation of their antimicrobial activity acting alone or in combined processes for food preservation. *Journal of the Science of Food and Agriculture*.

[33] Espina L., Somolinos M., Ouazzou A. A., Condón S., García-Gonzalo D., Pagán R. (2012). Inactivation of *Escherichia coli* O157: H7 in fruit juices by combined treatments of citrus fruit essential oils and heat. *International Journal of Food Microbiology*.

[34] Yousef A. E., Courtney P. D., Yousef A. E., Juneja V. K. (2003). Basics of stress adaptation and implications in new generation foods. *Microbial Stress Adaptation and Food Safety*.

[35] Miyamoto K. N., Monteiro K. M., da Silva Caumo K., Lorenzatto K. R., Ferreira H. B., Brandelli A. (2015). Comparative proteomic analysis of *Listeria monocytogenes* ATCC 7644 exposed to a sublethal concentration of nisin. *Journal of Proteomics*.

[36] Caballero Gómez N., Abriouel H., Ennahar S., Gálvez A. (2013). Comparative proteomic analysis of *Listeria monocytogenes* exposed to enterocin AS-48 in planktonic and sessile states. *International Journal of Food Microbiology*.

[37] Hanawa T., Fukuda M., Kawakami H., Hirano H., Kamiya S., Yamamoto T. (1999). The *Listeria monocytogenes* DnaK chaperone is required for stress tolerance and efficient phagocytosis with macrophages. *Cell Stress & Chaperones*.

[38] Pinto E., Marques N., W. Andrew P., Leonor Faleiro M. (2012). Over-production of P60 family proteins, glycolytic and stress response proteins characterizes the autolytic profile of *Listeria monocytogenes*. *Advances in Microbiology*.

[39] Periago P. M., Abee T., Wouters J. A. (2002). Analysis of the heat-adaptive response of psychrotrophic *Bacillus weihenstephanensis*. *International Journal of Food Microbiology*.

[40] Periago P. M., van Schaik W., Abee T., Wouters J. A. (2002). Identification of proteins involved in the heat-stress response of *Bacillus cereus* ATCC14579. *Applied and Environmental Microbiology*.

[41] Huang G., Mason S., Hudson J., Clerens S., Plowman J., Hussain M. (2014). Proteomic differences between *Listeria monocytogenes* isolates from food and clinical environments. *Pathogens*.

[42] Soni K. A., Nannapaneni R., Tasara T. (2011). The contribution of transcriptomic and proteomic analysis in elucidating stress adaptation responses of *Listeria monocytogenes*. *Foodborne Pathogens and Disease*.

[43] Melo J., Schrama D., Hussey S., Andrew P. W., Faleiro M. L. (2013). *Listeria monocytogenes* dairy isolates show a different proteome response to sequential exposure to gastric and intestinal fluids. *International Journal of Food Microbiology*.

[44] Begley M., Hill C. (2015). Stress Adaptation in foodborne Pathogens. *Annual Review of Food Science and Technology*.

